# Severe central and peripheral paraneoplastic demyelination associated with tumours of the ovaries

**DOI:** 10.1007/s00381-015-2731-5

**Published:** 2015-05-13

**Authors:** Elzbieta Jurkiewicz, Katarzyna Kotulska, Katarzyna Nowak, Katarzyna Malczyk, Julita Borkowska, Małgorzata Bilska

**Affiliations:** Department of Diagnostic Imaging, The Children’s Memorial Health Institute, Al. Dzieci Polskich 20, 04-730 Warsaw, Poland; Department of Neurology and Epileptology, The Children’s Memorial Health Institute, Al. Dzieci Polskich 20, 04-730 Warsaw, Poland

**Keywords:** Children, Magnetic resonance imaging, Brain, Paraneoplastic neurological symptom, Paraneoplastic demyelination, Ovarian teratoma

## Abstract

**Purpose:**

The aim of the study is to present MRI examinations of the brain and spinal cord, performed in girls with acute severe neurological presentation of paraneoplastic syndrome associated with ovarian teratomas. Paraneoplastic neurological syndrome (PNS) is a rare disorder caused by remote effects of malignancy in different organs. The pathogenesis of PNS concerns the autoimmune system and specific antibodies. PNS can be seen as encephalomyelitis, limbic encephalitis, progressive multifocal leukoencephalopathy, cerebellar ataxia, brainstem encephalitis, and paraneoplastic cerebellar degeneration. These symptoms are potentially reversible, if the underlying neoplasm is removed.

**Methods:**

We presented three girls, aged 13, 17, and 18 years. They were all referred to the hospital because of an acute onset of severe disseminated encephalomyelitis. All MRI exams were performed on a 1.5 T scanner with a routine brain and spinal cord protocol, including TSE T2-WI and FLAIR sequences. In all cases, a contrast agent was injected in the standard dose.

**Results:**

Neurological examination performed at the onset of the disease revealed hemiparesis, seizures, and consciousness disturbances. In one girl, visual field loss was also disclosed. They were all healthy before the onset of the disease. Brain and spinal cord MR imaging revealed multiple hyperintense lesions located supratentorially in the white matter of both hemispheres, in the pons, cerebellum, and spinal cord. Patients were treated with methyloprednisolone IV and IVIG. They all improved but significant sequelae were present. Two of them developed symptoms of acute demyelinating polyradiculoneuropathy within 2 months after the onset of encephalomyelitis. At the same time, brain MRI showed progression of the lesions. In two patients, anti-Yo antibodies were present in blood. Extensive examinations revealed bilateral ovarian teratomas in two patients, and left-sided ovarian teratoma in one case. Surgical resection of teratomas resulted in rapid clinical improvement.

**Conclusions:**

These cases show that in children and adolescents, acute demyelinating disease can be a manifestation of paraneoplastic neurological syndrome. Thus, PNS should always be considered in the differential diagnosis of encephalomyelitis. In female children and adolescents with suspected PNS, it is important to search for ovarian tumours.

## Introduction

Paraneoplastic neurological syndromes (PNSs) are rare disorders caused by the remote effects of tumour cells on the nervous system. PNSs may be the first manifestation of a neoplasm and can develop before the diagnosis of a neoplasm is made in 80 % of cases [1]. Most cases of PNS are reported in adults or the elderly. Incidence in children is unknown [2].

The pathogenesis of PNS is associated with immune cross-reactivity between tumour cells and nervous system. It has been demonstrated that patients suffering from PNS produce onconeural antibodies [3]. The hypothesis of an immune-mediated response against the nervous system is the currently accepted explanation of this pathogenic syndrome [4, 5]. In the brain, PNS affects mainly limbic structures and brainstem, but any brain region can be affected. The peripheral nervous system can also be affected. PNS can present as encephalomyelitis, limbic encephalitis, progressive multifocal leukoencephalopathy, cerebellar ataxia, brainstem encephalitis or paraneoplastic cerebellar degeneration. The most common PNSs affecting the central nervous system detected by magnetic resonance imaging are limbic encephalitis, encephalomyelitis and subacute cerebellar ataxia. PNSs are primarily caused by a small-cell lung cancer, followed by gastrointestinal and genitourinary cancers (ovary, kidney, uterus), Hodgkin’s lymphoma, breast cancer and testicular cancer [6]. In children, PNSs are most frequently associated with neuroblastoma, teratoma of various location and Hodgkin’s lymphoma. These symptoms are potentially reversible as long as the underlying neoplasm is removed [2, 5].

Due to its high sensitivity, magnetic resonance imaging (MRI) of the brain and spinal cord is particularly useful in showing abnormal findings early in the course of the disease.

We reported on MRI examinations of the brain and spinal cord performed in three girls with acute severe neurological paraneoplastic syndrome associated with ovarian teratomas.

## Material and methods

Three girls, aged 13, 17, and 18 years, were referred to our hospital because of an acute onset of severe disseminated encephalomyelitis. They were all healthy before the onset of the disease. In all cases, the clinical course of the disease, the results of immunological tests, and radiological imaging of the brain, spinal canal, chest, abdomen and pelvis, were analysed.

All MRI exams were performed using a 1.5 T scanner with routine brain and spinal cord protocol. MRI of the brain was performed using an 8-channel phased-array head coil. The examination protocol included axial TSE T2WI, and FLAIR sequences, sagittal and coronal TSE T2WI, sagittal and coronal fl T1WI. MRI of the spinal cord was performed using a spinal phased-array coil. The MRI protocol consisted of T1-, T2-weighted sagittal and axial images visualising the entire length of the spinal cord.

T1-weighted images were performed before and after contrast injection with a routine dose of 0.1 mmol/kg Gd-DTPA.

## Results

### Clinical findings

Neurological examination performed at the onset of the disease revealed symptoms of severe encephalitis in all girls, including hemiparesis, seizures, visual loss and consciousness disturbances. Detailed clinical presentation in each of the patients is presented in Table 1.

**Table 1:** Clinical presentation and laboratory findings

	Case 1	Case 2	Case 3
Sex/age	F/13	F/17	F/18
Clinical findings			
Hemiparesis	Left, grade II	Left, grade I	Right, total
Seizures	Focal and generalised	Focal	No
Consciousness disturbances	Unconsciousness	Somnolence and hallucinations	Somnolence
Visual loss	Partial	No	Yes, total in the left eye
Subsequent symptoms	Severe acute demyelinating polyneuropathy two weeks after the onset of encephalitis	After partial recovery, next relapse of encephalitis three weeks after the first event	Severe acute demyelinating polyneuropathy four weeks after the onset of encephalitis
Laboratory findings			
CSF	Abnormal	Abnormal	Abnormal
Increased pleocytosis	Yes	Yes	Yes
Increased protein concentration	Yes	Yes	Yes
Oligoclonal bands	No	No	Yes
Anti-Yo antibodies	No	Yes	Yes

In all three cases, the onset of clinical symptoms was acute. In case 1, sudden morning paresis of the upper and lower limbs with bulbar paralysis occurred. In case 2, the initial symptoms included temperature above 39 °C, voiding disturbances, followed by consciousness disturbances and left hemiparesis on the next day. In case 3, sudden weakness of the upper and lower limbs occurred; the patient was treated with intravenous immunoglobulin due to suspicion of Guillain-Barre syndrome. After a period of 2 weeks, visual disturbances in the right eye, consciousness disturbances and left hemiparesis appeared.

In each case, urgent hospitalisation was required. Clinical symptoms that were later observed during hospitalisation are shown in Table 1.

Severe acute demyelinating polyneuropathy, confirmed by electrophysiological examination, was observed in two patients at week 2 (case 1) and week 4 (case 3) following the onset of encephalomyelitis.

### Laboratory findings

Routine laboratory results were normal in all girls, except for an elevated sedimentation rate (56/h) in case 3. The CSF was abnormal in all patients, with increased pleocytosis (50/mm3, 89/mm3, and 140/mm3 in case 1, 2, and 3, respectively) and increased protein concentration (95, 86, and 250 mg/dL in case 1, 2, and 3, respectively). In one patient (case 3), oligoclonal bands were present in the CSF.

A second CSF examination was performed in the two girls (case 1, 3) who developed symptoms of acute polyneuropathy. It revealed slight pleocytosis and significantly increased protein concentration. Electrophysiological examination revealed features of demyelinating polyneuropathy in both girls.

In two girls (case 2, 3), onconeuronal anti-Yo antibodies were present in the blood. Laboratory findings are presented in Table 1.

### Imaging findings

#### Brain MR imaging

**Fig. 1:**
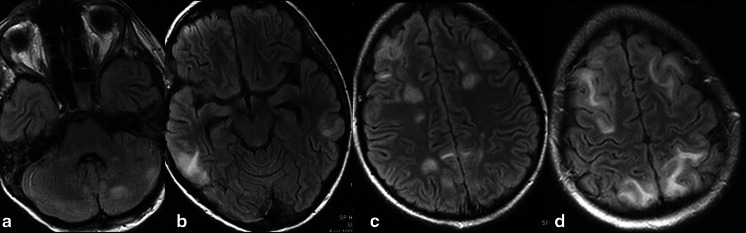
Case 1. **a**–**d** Axial T2-FLAIR images of the brain with hyperintensities in both cerebral hemispheres and the left cerebellar hemisphere

MRI examinations revealed symmetric and asymmetric hyperintensity on T2-weighted images and FLAIR sequences in the temporal and frontal lobes with cortical and subcortical white matter involvement. The semioval center was involved in all three cases. Additionally, in case 1, both parietal lobes and the left occipital lobe were involved (Fig. 1). Multiple lesions in the left cerebellar hemisphere were also observed in cases 1 and 3. In one girl (case 2), additional lesions in the pons and left occipital lobe were observed. Diffusion restriction was observed in cases 1 and 2. Enhancement was seen in two patients (case 2, 3).

#### Spinal cord MR imaging

**Fig. 2:**
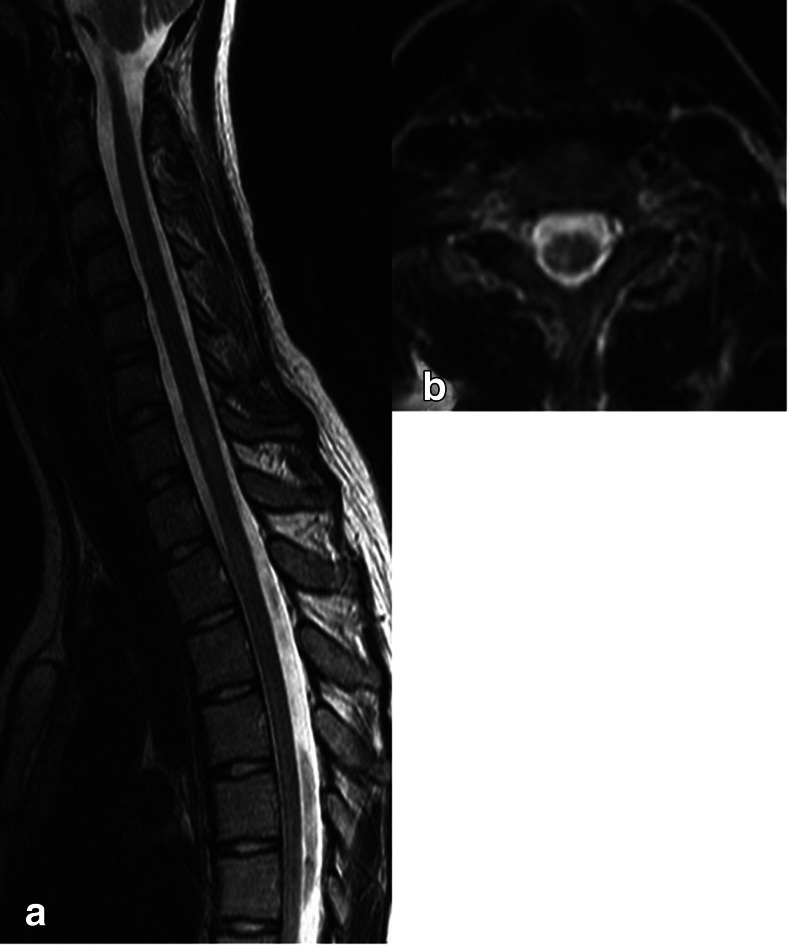
Case 2. **a**. Sagittal T2-weighted image of the spinal cord demonstrates two lesions located on C7, and Th2 levels. **b**. axial T2-weighted image shows hyperintense lesions located on the right side of the thoracic spinal cord

Spinal cord lesions were found in a 17-year-old girl (case 2). They were located at levels C7, Th2 and Th3 (Fig. 2). No spinal cord lesions were found in case 1. One patient (case 3) had no magnetic resonance imaging of the spinal canal.

**Fig. 3:**
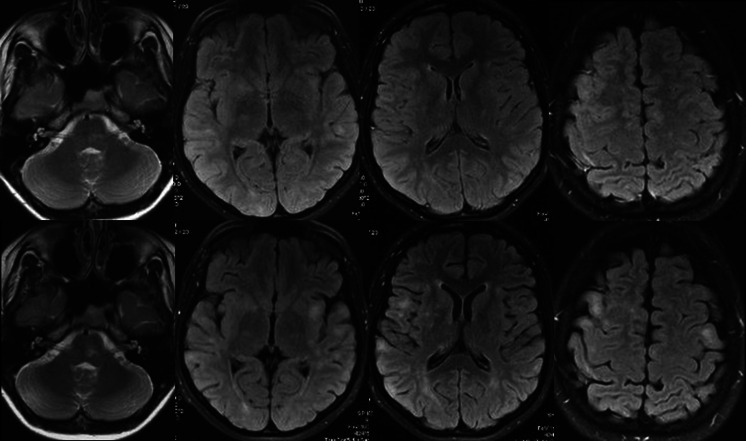
Axial T2- and T2-FLAIR images, two MRI examinations in case 2. *Bottom row* shows progression of lesions seen after 2 weeks

Follow-up brain MRI examinations performed after 2 weeks and after 2 months, before the correct diagnosis and proper treatment were established, showed progression of the lesions in two patients (case 2 and 3) (Fig. 3).

Magnetic resonance findings are summarized in Table 2.

**Table 2:** MR imaging of the lesions in the brain and spinal cord

	Case 1	Case 2	Case 3
Sex/age	F/13	F/17	F/18
Supratentorial subcortical white matter	Yes	Yes	Yes
Supratentorial deep white matter	Yes	Yes	Yes
Supratentorial grey matter	Yes	Yes	Yes
Corpus callosum	No	No	Yes
Pons	No	Yes	Yes
Medulla oblongata	No	No	Yes
Cerebellum	Yes	No	Yes
Diffusion restriction	Yes	Yes	Not performed
Enhancement	No	Yes	Yes
Progression	No	Yes	Yes
Spinal cord	No	Yes	Not performed

**Fig. 4:**
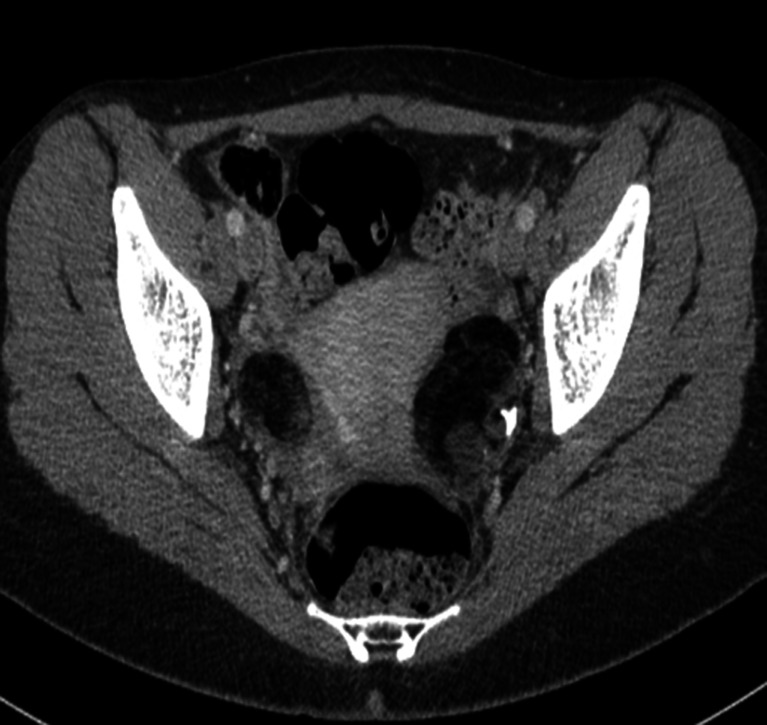
Case 2. Axial unenhanced CT scan demonstrates bilateral teratomas of the ovaries with intratumoral fat and focal calcifications (seen on the *left side*)

Ultrasound examinations and computed tomography scans (Fig. 4) revealed bilateral teratomas in two patients and a left-sided ovarian teratoma in one patient (case 3). Surgical resection of the neoplasms resulted in rapid clinical improvement in all three girls. A histopathological examination confirmed the tumours to be benign ovarian cystic teratomas.

The final diagnosis was made after 4 months of clinical symptoms duration in case 1 and after 2 months in cases 2 and 3.

Rapid clinical improvement was observed in all three cases just days after the introduction of proper treatment, with further improvement observed over the following weeks. In case 1, the final neurological status includes minimal foot drop with no gait disturbances. In case 2, the neurological examination is normal. In case 3, amblyopia on the right and left hemiparesis grade 1 are still observed.

## Discussion

Paraneoplastic syndrome is an uncommon disorder that is likely underreported due to difficulty in establishing the diagnosis [7]. Diseases with infectious etiology, such as herpes simplex virus encephalitis, have similar clinical and MR imaging features. Neurological symptoms usually develop before the diagnosis of the tumour. Paraneoplastic encephalomyelitis is characterized by involvement of different areas of the brain hemispheres, brainstem, cerebellum, and spinal cord with different distribution of the disease [8, 9].

All of our patients presented symptoms of encephalomyelitis. MRI examinations revealed a rare combination of paraneoplastic limbic encephalitis, brainstem, and spinal cord involvement: multiple lesions located supra- and infratentorially, and in the spinal cord. As in the previously published cases, we observed hyperintense lesions involving the cortex and subcortical white matter of the brain hemispheres [10–13]. The lesions were detected on FLAIR sequences and on T2-weighted images. In one girl (case 2), we observed a lesion in both pons and spinal cord; in remaining two (case 1 and 3), in the cerebellum. Some of the lesions revealed restricted diffusion. We did not observe signs of haemorrhage. Contrast enhancement was present in two patients. Typical MRI appearance of encephalomyelitis includes involvement of the grey and white matter, regardless of etiology and the absence of haemorrhage. It may be difficult to find the etiology of encephalomyelitis in such cases. In all three of our patients, ovarian teratomas were recognized. The association of neurological symptoms in patients with ovarian teratoma was previously reported [10–13].

In order to diagnose PNSs, it is necessary to identify antionconeuronal protein antibodies in the patient’s serum. However, they are present in only 50–60 % of cases [6, 14]. PNS-specific paraneoplastic antibodies include anti-Yo, anti-Hu, anti-CV2, anti-Tr, anti-metabotropic glutamate receptor type 1, and anti-Ri. Anti-Yo antibodies may be associated with cancers of the ovary, endometrium, and breast, as well as paraneoplastic cerebellar degeneration [15, 16]. Anti-Yo antibodies were found in two of our patients (cases 2 and 3), without the features of cerebellar degeneration. Benign ovarian cystic teratomas were found in all of our patients.

Although an association between PNSs and antionconeuronal antibodies has been well established, there is no definitive proof of any specific cause-effect relationship between the presence of antibodies and the development of a clinical syndrome [6, 17].

## Conclusions

It is extremely important to remember that paraneoplastic neurological syndrome can be the first manifestation of a malignancy, and that neurological symptoms usually develop before the diagnosis of a tumour. Paraneoplastic neurological syndrome should be considered in the differential diagnosis of encephalomyelitis. In female children and adolescents, the ovarian tumour is one of the most commonly associated neoplasms.
